# Automated Hierarchy Evaluation System of Large Vessel Occlusion in Acute Ischemia Stroke

**DOI:** 10.3389/fninf.2020.00013

**Published:** 2020-03-24

**Authors:** Jia You, Anderson C. O. Tsang, Philip L. H. Yu, Eva L. H. Tsui, Pauline P. S. Woo, Carrie S. M. Lui, Gilberto K. K. Leung

**Affiliations:** ^1^Department of Statistics and Actuarial Science, The University of Hong Kong, Hong Kong, Hong Kong; ^2^Division of Neurosurgery, Department of Surgery, The University of Hong Kong, Hong Kong, Hong Kong; ^3^Department of Statistics and Workforce Planning, Hospital Authority, Hong Kong, Hong Kong

**Keywords:** acute ischemic stroke, large vessel occlusion, prognosis, machine learning, deep learning

## Abstract

**Background:**

The detection of large vessel occlusion (LVO) plays a critical role in the diagnosis and treatment of acute ischemic stroke (AIS). Identifying LVO in the pre-hospital setting or early stage of hospitalization would increase the patients’ chance of receiving appropriate reperfusion therapy and thereby improve neurological recovery.

**Methods:**

To enable rapid identification of LVO, we established an automated evaluation system based on all recorded AIS patients in Hong Kong Hospital Authority’s hospitals in 2016. The 300 study samples were randomly selected based on a disproportionate sampling plan within the integrated electronic health record system, and then separated into a group of 200 patients for model training, and another group of 100 patients for model performance evaluation. The evaluation system contained three hierarchical models based on patients’ demographic data, clinical data and non-contrast CT (NCCT) scans. The first two levels of modeling utilized structured demographic and clinical data, while the third level involved additional NCCT imaging features obtained from deep learning model. All three levels’ modeling adopted multiple machine learning techniques, including logistic regression, random forest, support vector machine (SVM), and eXtreme Gradient Boosting (XGboost). The optimal cut-off for the likelihood of LVO was determined by the maximal Youden index based on 10-fold cross-validation. Comparisons of performance on the testing group were made between these techniques.

**Results:**

Among the 300 patients, there were 160 women and 140 men aged from 27 to 104 years (mean 76.0 with standard deviation 13.4). LVO was present in 130 (43.3%) patients. Together with clinical and imaging features, the XGBoost model at the third level of evaluation achieved the best model performance on testing group. The Youden index, accuracy, sensitivity, specificity, F1 score, and area under the curve (AUC) were 0.638, 0.800, 0.953, 0.684, 0.804, and 0.847, respectively.

**Conclusion:**

To the best of our knowledge, this is the first study combining both structured clinical data with non-structured NCCT imaging data for the diagnosis of LVO in the acute setting, with superior performance compared to previously reported approaches. Our system is capable of automatically providing preliminary evaluations at different pre-hospital stages for potential AIS patients.

## Introduction

Acute ischemic stroke (AIS) is a leading cause of morbidity and mortality worldwide, and it is usually due to a focal interruption of cerebral blood flow caused by occlusion of a cerebral artery. Large vessel occlusion (LVO) accounts for approximately only one-third of AIS but is responsible for 60% of stroke-related disability and 90% of stroke-related deaths (Malhotra et al., 2017). Recent advances in endovascular thrombectomy (EVT) for treatment of AIS caused by LVO have been widely accepted around the world (Powers et al., 2019). Similar to intravenous thrombolysis, rapid access to EVT, preferably within 6 h from symptom onset, remains paramount to ensure functional recovery (Saver et al., 2016; Powers et al., 2019). As EVT is only available in specialized centers, interhospital transfer is frequently required, leading to an average treatment delay of 142 min and millions of neuron loss (Saver, 2006; Saver et al., 2016). Prehospital care therefore focuses on rapid identification of AIS and direct transport to a hospital ideally suited to care for that patient, avoiding the lengthy time delays of interfacility transfers (Prabhakaran et al., 2011).

Recent decades have witnessed the development of prehospital LVO prediction scales in order to differentiate LVO from milder strokes, allowing paramedics to make rapid diagnosis in the prehospital setting. Popular scales include the three-item Stroke Scale (3I-SS) (Singer et al., 2005), the Los Angeles Motor Scale (LAMS) (Nazliel et al., 2008), the Rapid Arterial Occlusion Evaluation (RACE) Scale (Pérez de la Ossa et al., 2014), the Cincinnati Prehospital Stroke Severity (SS) Scale (Katz et al., 2015), the Field Assessment Stroke Triage for Emergency Destination (FAST-ED) (Lima et al., 2016), and the Prehospital Acute Stroke Severity (PASS) (Hastrup et al., 2016). Some of these scales specifically aim to identify stroke patients with LVO rather than all AIS patients. These scales are simplified from National Institutes of Health Stroke Scale (NIHSS) items, a 42-point criterion standard for stroke, and then transformed with different linear combinations based on the correlation between patients’ clinical symptoms and the presence of stroke. The drawbacks of these measurements are the ignorance of patients’ potential stroke-related co-morbidities and risk factors, such as age and clinical history. Moreover, non-linear relationship was not considered. In addition, the level of training of paramedical staff will affect the utility of these scales that rely on physical examination.

Compared with the previous standard scales, this study intends to construct an automated LVO ischemic stroke evaluation system based on a data hierarchy of patients’ symptoms from onset to final diagnosis. There are three stages of modeling in this evaluation system. The first stage (Level-1) attempts to aid prehospital triage during initial patient contact by the emergency dispatch staff, using only basic demographic information and easily observed symptoms (Harbison et al., 2003); while the second stage (Level-2) aims to have a fast but more accurate assessment using additional pre-existing clinical features and vital signs available on ambulance or at emergency departments; the last stage (Level-3) involves patients non-contrast CT (NCCT) scans to further enhance the evaluation to assist EVT pathway activation.

Given the LVO label for each patient, all three level models were implemented with supervised learning. This algorithm is capable to establish an approximate function that maps an input to an output based on example input-output pairs (Russell and Norvig, 2016). Hence, when new input comes in, the function can automatically give predictions. There are a variety of machine learning techniques for the approximate mapping functions. This study applied multiple popular machine learning algorithms, namely, logistic regression, random forest, support vector machine (SVM), and eXtreme Gradient Boosting (XGBoost). Comparisons between these methods are explored to validate our LVO evaluation system.

Additionally, for the usage of NCCT brain data in the Level-3 model, deep learning (LeCun et al., 2015) was adopted due to its state-of-the-art performances in many computer vision tasks during the past several years. There are multiple applications in the medical imaging domain (Litjens et al., 2017), such as diagnosis classification (Esteva et al., 2017), cancer detection (Cireşan et al., 2013), and lesion segmentation (Havaei et al., 2017). In this study, we adopted deep learning model as a feature extractor of brain NCCT scans in Level-3 model.

## Materials and Methods

### Study Population and Data Acquisition

The patients within the database were retrospectively stratified using a disproportionate random sampling method from the Hong Kong Hospital Authority’s clinical management system (HACMS) at year 2016. This database holds records of all patients admitted to the public hospitals, including their demographic and clinical profiles, diagnoses, treatment procedures, and outcomes. Patients who met the following inclusion criteria were chosen: (a) over 18 years old; (b) with a principal diagnosis coding of AIS; (c) admitted via Accident and Emergency Department (AED); and (d) with an NCCT brain scan performed within 24 h of AED admission. The pre-existing chronic diseases of the study subjects were defined and extracted based on the Chronic Diseases Virtual Registry for all patients ever treated in the public hospital system.

A total of 300 subjects were selected and were randomly split into 200 for model training and 100 for model testing. The data used in this study were of various types, e.g., basic demographic data, clinical data (including pre-existing medical conditions, blood test parameters, and vital signs), and corresponding NCCT scans. Feature details can be found in Tables 1A,B. All patients had their corresponding brain NCCT scans as well. Those scans had similar quality, spatial resolution, and field-of-view. The in-plane resolution was 0.426^∗^0.426 mm. The slice thickness is 5.0 mm for most cases. Each axial slice has identical size of 512^∗^512 pixels.

**TABLE 1:** **(A,B)** Level-1 and Level-2 features summary statistics and *P*-values.

		LVO 130	w/o LVO 170	*p*-Value
**(A)**				
Age	Mean	79.69	73.19	**2.00e-05**
	95% CI	77.55–81.84	71.15–75.24	
Gender	Female	84(64.6%)	76(58.5%)	**7.05e-04**
	Male	46(35.4%)	94(41.5%)	
Limb weakness	Yes	129(99.2%)	126(74.1%)	**2.84e-11**
	No	1(0.8%)	44(25.9%)	
Left limb weakness	Yes	59(45.4%)	63(37.1%)	1.56e-01
	No	71(54.6%)	107(62.9%)	
Right limb weakness	Yes	73(56.2%)	70(41.2%)	1.06e-02
	No	57(43.8%)	100(58.8%)	
Facial weakness	Yes	41(31.5%)	44(25.9%)	**1.06e-05**
	No	11(8.5%)	53(31.2%)	
	Unknown	78 (60%)	73(42.9%)	
Left facial weakness	Yes	19(14.6%)	25(14.7%)	**5.74e-03**
	No	33(25.4%)	72(42.4%)	
	Unknown	78 (60%)	73(42.9%)	
Right facial weakness	Yes	22(16.9%)	19(11.2%)	**2.39e-04**
	No	30(23.1%)	78(45.9%)	
	Unknown	78 (60%)	73(42.9%)	
Speech deficits	Yes	62(47.7%)	72(42.4%)	4.12e-01
	No	68(52.3%)	98(57.6%)	
**(B)**				
Glasgow Coma Scale (GCS)	Mean	10.70	13.68	**<2.20e-16**
	95% CI	10.23–11.17	13.28–14.09	
	NA	9(6.9%)	15(8.8%)	
GCS_Eye	Mean	3.18	3.83	**6.69e-07**
	95% CI	2.96–3.41	3.72–3.93	
	NA	32(24.6%)	25(14.7%)	
GCS_Verbal	Mean	2.44	4.25	**<2.20e-16**
	95% CI	2.14–2.74	4.02–4.48	
	NA	32(24.6%)	25(14.7%)	
GCS_Motor	Mean	5.16	5.70	**2.44e-05**
	95% CI	4.96–5.36	5.56–5.85	
	NA	32(24.6%)	25(14.7%)	
Diastolic blood pressure	Mean	84.50	83.90	8.04e-01
	95% CI	80.71–88.29	80.99–86.82	
	NA	56(43.1%)	78(45.9%)	
Systolic blood pressure	Mean	154.82	162.40	5.90e-02
	95% CI	148.62–161.02	157.46–167.34	
	NA	56(43.1%)	78(45.9%)	
Diabetes mellitus	Yes	30(23.1%)	45(26.5%)	5.91e-01
	No	100(76.9%)	125(73.5%)	
Hypertension	Yes	89(68.5%)	118(69.4%)	9.0e-01
	No	41(31.5%)	52(30.6%)	
Smoker	Yes	15(11.5%)	47(27.6%)	**2.63e-03**
	No	62(47.7%)	70(41.2%)	
	Unknown	53(40.8%)	53(31.2%)	
Current smoker	Yes	8(6.2%)	22(12.9%)	6.66e-02
	No	69(53.1%)	95(55.9%)	
	Unknown	53(40.8%)	53(31.2%)	
EX smoker (quitted > 2 years)	Yes	7(5.4%)	25(14.7%)	1.91e-02
	No	70(53.8%)	92(54.1%)	
	Unknown	53(40.8%)	53(31.2%)	
Atherosclerosis	Yes	3(2.3%)	6(3.5%)	2.31e-02
	Unknown	127(97.7%)	164(96.5%)	
Atrial fibrillation	Yes	48(36.9%)	32(18.8%)	**7.22e-04**
	Unknown	82(63.1%)	138(81.2%)	
Cardiombolism	Yes	8(6.2%)	2(1.2%)	3.98e-02
	Unknown	122(93.8%)	168(98.8%)	
Valvular heart disease	Yes	61(46.9%)	86(50.6%)	6.08e-01
	Unknown	69(53.1%)	84(49.4%)	

*Continuous variables: means, 95% confidence intervals, number of missing values and their proportions, p-values were calculated through Student’s t-test. Discrete variables: contingency tables with figures and proportions, p-values were calculated through Fisher’s exact test (2 × 2 tables) or Pearson’s Chi-square test (multiple rows and columns tables). Boldfaced p-values indicated strong dependencies based on 1% significance level.*

The diagnosis of anterior circulation LVO was independently verified by two cerebrovascular disease specialists (>8 years experiences in interpretation of acute stroke neuroimaging) based on available admission notes, discharge notes, and CT scans on admission and 24–72 h after stroke onset. Those with severe stroke (NIHSS > 8) and corresponding large infarct in the ICA or MCA territories on the follow-up CT were considered LVO when no CT angiogram was available at presentation (Demeestere et al., 2017). Any discrepancies were resolved by consensus. Besides, the presence of hyperdense middle cerebral artery (MCA) sign on NCCT was verified by two cerebrovascular disease specialists and manually drawn through FMRIB Software Library (FSL) (Smith et al., 2004; Jenkinson et al., 2012). This sign is a direct visualization of thromboembolic material within the vessel lumen, which has been reported as a specific and important sign for intravascular thrombus in the diagnosis of AIS and LVO (Gasparian et al., 2015).

### Hierarchical Modeling

**FIGURE 1:**
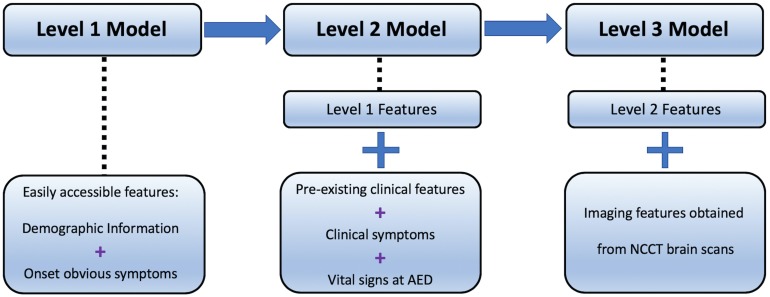
Hierarchy data of Level-1, -2, and -3.

The study aimed to develop machine learning models for LVO prediction based on a data hierarchy of three different levels (Figure 1).

Level-1 model utilized patients’ demographic information, e.g., age and gender, and some basic symptoms that can be easily observed even by lay-persons, including the presence of speech deficits, facial weakness, left- and right-sided facial weakness, limb weakness, and left- and right-sided limb weakness.

In addition to the features used in Level-1, Level-2 involved clinical data, consisting of the pre-existing medical conditions such as diabetes mellitus and hypertension, whether the patient was a smoker, current smoker (or quitted = 2 years), previous smoker (quitted > 2 years), diastolic and systolic blood pressure, Glasgow Coma Scale (GCS), and its corresponding sub-scales of eye, verbal, and motor function. Prior diagnoses of atrial fibrillation, atherosclerosis, cardiombolism, and valvular heart disease were included as well.

Together with the structured data in Level-1 and Level-2 models, image features obtained from CT scans served as additional information in Level-3 model. Hence, the deep learning architecture in this step worked as a feature extractor that converts non-structured imaging data into encoded structured features.

### Multiple Machine Learning Algorithms

Binary logistic regression is used to predict the odds which is defined as the probability of an event happened divided by the probability that the event not happened. The advantages of the logistic regression are its simplicity, fast training speed, and the widely use of log odds for investigating the relative risk of various predictors on the binary outcome. However, the model does not allow missing data and it cannot detect a non-linear structure automatically and adaptively inherited the non-linear structure in the model.

Random forest is an ensemble method which aims to enhance the model performance by combining many weak classifiers such as decision trees. Given a training set, random forest first generates many bootstrap samples as the training set. Then a decision tree is built for each bootstrap sample using a subset of predictors randomly selected to consider splitting in each node. Finally, taking the average of the predicted probabilities of the binary outcome obtained from these fitted trees gives the predicted probability for the fitted random forest. Random forest models can be trained fairly quickly because of the inherent parallel computing. Besides, unlike other machine learning models, its randomness avoids the training to get stuck at a local minimum; hence, it can be made more complex to improve the prediction accuracy without the risk of overfitting.

Support vector machine takes each data point as a vector in *m*-dimensional space (where *m* is the number of variables) with the value of each variable being the value of a particular coordinate. Then, it is capable to differentiate different classes by identifying the hyper-plane. It is not hard to find a linear hyper-plane between two classes; however, many cases are non-linear. The most significant benefit of SVM comes from the fact that they are not restricted to being linear classifiers, where it contains functions that can take low dimensional input space and transform it to a higher dimensional space, hence the algorithm become much more flexible by introducing various types of non-linear decision boundaries.

The XGBoost is an efficient and scalable implementation of gradient boosting framework by J. Friedman (Friedman, 2002; Chen and Guestrin, 2016). XGBoost is now a widely used and popular machine learning technique among data scientists’ communities. It is an ensemble technique that builds the model in a stage-wise method that new models are added to correct the errors made by the previously trained models. New models are added sequentially until no further improvement can be made. It is a highly flexible and versatile approach that can work through most regression, classification, and ranking tasks as well as customized objective functions.

### Deep Learning Feature Extractor

**FIGURE 2:**
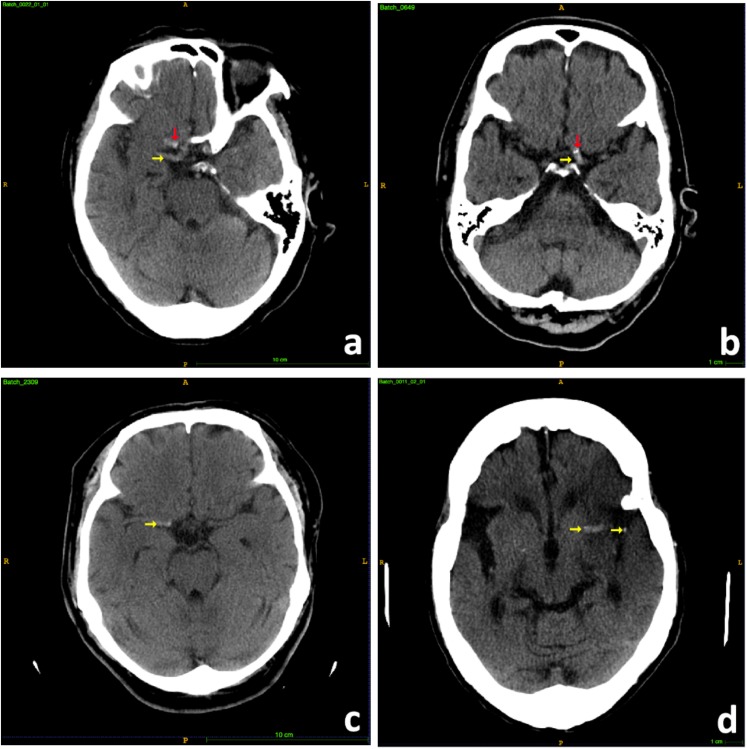
Samples of hyperdense MCA signs. **(a)** Right hyperdense MCA sign (yellow arrow), partial volume artifact of bony anterior clinoid process (red arrow). **(b)** Left hyperdense MCA sign (yellow arrow), MCA calcifications (red arrow). **(c)** Right hyperdense MCA sign (yellow arrow). **(d)** Left hyperdense MCA sign (yellow arrow).

The hyperdense MCA sign is a high attenuation blood clot within the MCA on CT scans and can be identified as irregular brighter dot comparing with surrounding textures due to focal increased density (Figure 2). The hyperdensity served as a significant biomarker for large thromboembolic occlusion and is highly correlated with LVO ischemia stroke (Lim et al., 2018). Therefore, it can be inferred that features from a well-trained MCA sign segmentation architecture can be helpful in the prediction of LVO. Thus, we built a model in order to segment the MCA sign and applied the model as a feature extractor to obtain useful features from CT scans.

#### Non-contrast CT Pre-processing

**FIGURE 3:**
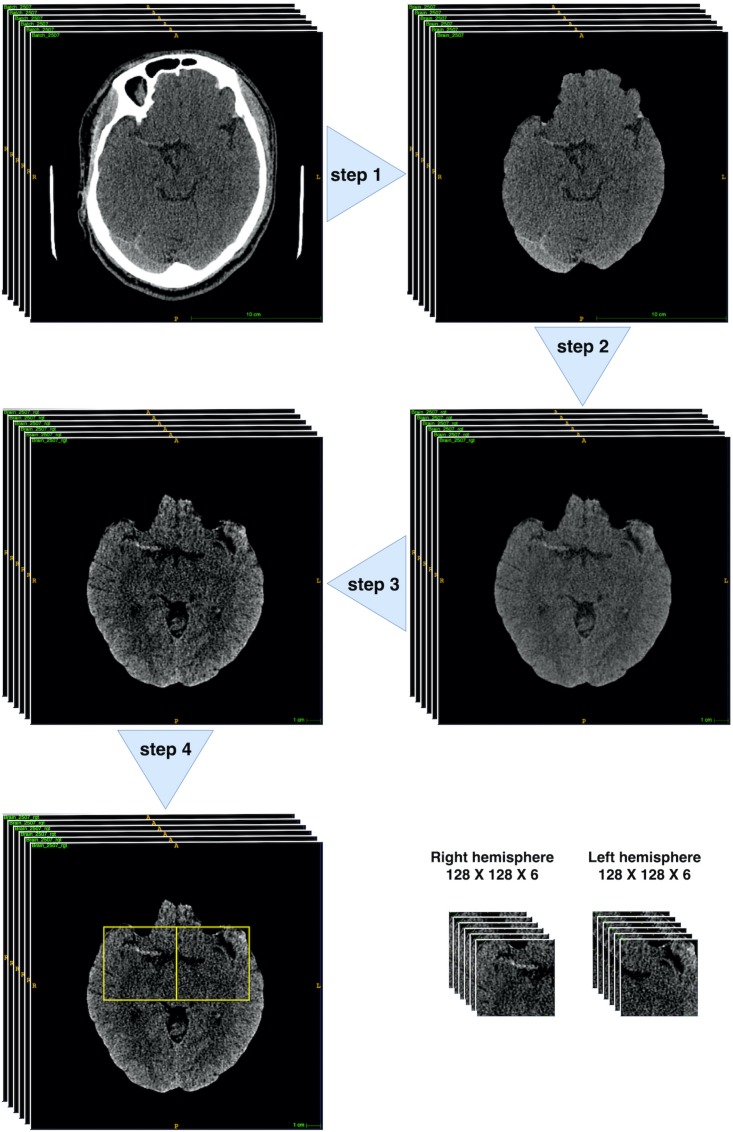
Brain CT pre-process workflow. Step 1: brain extraction. Step 2: 2D rigid registration. Step 3: Median filter and intensity thresholding to [20, 80]. Step 4: bounding box extraction (through 4th–10th slices with two symmetric bounding boxes cropped within both hemispheres ([128: 256, 212:340] and [256: 384, 212:340]) on the 512 × 512 pixelwise CT scan). All pre-processing steps were implemented through *fsl* interface of package *nipype* under *Python.*

The MCA is located within the proximal Sylvian fissure near the center of the brain, posterior to the lesser wing of sphenoid bone, an anatomical landmark of the skull base. Hence, extraction of this region of interest (ROI) could allow the model to focus on specific candidate areas and largely eliminate irrelevant information. To have ROI extracted, we developed a fully automated pre-processing pipeline (Figure 3) through *fsl* interface of package *nipype* under *Python.*

There were mainly four steps within the pipeline: Step 1: skull stripping to extract the brains; Step 2: rigid-body 2D registration to a common template in order to ensure the brains were horizontally symmetrical and aligned; Step 3: median filter and thresholding to intensity [20, 80] to enhance the contrast of MCA signs; Step 4: Bounding box extraction through 4th–10th slices with two symmetric bounding boxes cropped within both hemispheres ([128: 256, 212:340] and [256: 384, 212:340]) on the 512 × 512 pixelwise CT scan. Finally, each patient would obtain 12 128 × 128 sized scans.

#### Deep Learning Architecture

**FIGURE 4:**
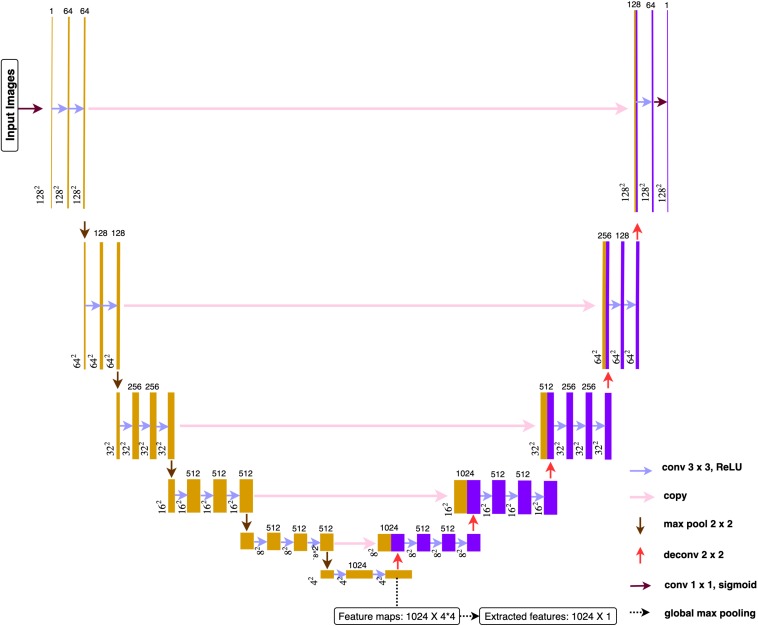
Deep learning architecture. All the convolution layers used 3 × 3 as size for receptive field and 1 for the stride. Paddling was applied to make sure all the feature maps had the identical size before and after convolutions. All activation function used *ReLu* after each convolution.

The proposed architecture (Figure 4) belonged to the category of fully convolutional networks (FCNs) (Long et al., 2015) that extended the convolution process across the entire image and predicts the segmentation mask from end-to-end. The architecture mainly contained two parts, encoding and decoding, where the encoding part extracted the image features from low to high complexity, while the decoding part transformed the features and reconstructed the segmentation label map from coarse to fine resolution. Besides, the model contained skip connections from encoding part to decoding part that able to make up the spatial detail lost during up-sampling. The overall architecture resembled U-net model (Ronneberger et al., 2015), an architecture especially focused on medical imaging segmentation tasks. A global max pooling layer was connected at the end of encoding part, where total 1024 feature maps, each with size 4^∗^4, converted to 1024 features. The encoding part pulled out image features from low to high intricacy, while the global max pooling layers grabbed the maximum representation from each feature map.

Only one slice per person would be selected to sever as image representation for feature extraction. If predicted segmentation exists, the slice with the largest predicted segmentation would be chosen. If no predicted segmentations, the middle slice (third) would be chosen. After feeding the representation image into the pre-trained deep learning model, 1024 features were obtained for each patient. Based on whether the patient suffered from LVO, features can be divided into two subgroups and conducted with two-sample *t*-test in order to do a brief filtering. Finally, top-10 image features with the smallest *p*-values were selected and combined with Level-2 features to build the whole feature set for Level-3.

#### Experiments

The deep learning model was trained with Adam optimizer with initial learning rate 1e-05 and momentum 0.9. To tackle the positive versus negative ground truth imbalance issue, Tversky loss (Salehi et al., 2017) and hard-negative-mining technique (Shrivastava et al., 2016) were adopted to tackle the data imbalance issue. The deep learning model was constructed through *tensorflow 1.13.1* and *keras 2.2.4* under *Python* and was trained for 200 epochs on Tesla K80 GPU card with batch size of 16.

### Missing Values

In the present study, there were no missing values within features in Level-1, but the pre-existing medical conditions in Level-2 contain some missing values. XGBoost was capable to handle missing values automatically during training, and it could also provide a robust prediction when observations contained incomplete features. In contrast, other machine learning methods such as logistic regression, random forest, and SVM do not allow missing data. Hence, data imputation was required for these algorithms.

For any categorical attributes containing missing values, a missing parameter “Unknown” was assigned to form a new category. For continuous attributes containing missing values, K-nearest-neighbors (KNN) imputation method (Crookston and Finley, 2008) was adopted. The principle idea was easy to follow. Given an observation containing a missing value in a continuous attribute *X*, the *k* nearest neighbor subjects in the training data were identified based on the Euclidean distance. The missing value was then imputed by the median of the non-missing *X* values among the *k* nearest neighbors. The KNN imputation method was easy to be deployed to the testing data.

### Variables Selection

Variable selection is the process of selecting a subset of relevant features for use in model building. It helps to simplify the models and avoids the overfitting by removing redundant or irrelevant features without loss of useful information.

Stepwise logistic regression selects a reduced number of predictor variables during the model building process by keeping adding significant features and removing insignificant features one at a time to find the best-fit logistic regression model.

Random forest and XGBoost can automatically select features while building trees. To fairly identify the relative importance of a set of feature variables, both tree-based methods adopt impurity-based ranking method to calculate variable importance scores. When growing a tree, it is required to compute the amount of the weighted impurity of each feature dropped in a tree. Then, for a forest, the impurity dropped for each feature can be averaged, which allows all features to be ranked and compared to each other.

The performance of SVM largely depends on the features since redundant variables have significant impact when constructing separable hyper-planes. However, SVM itself does not possess the ability to select variables. Thus, features selected from other algorithms would be used in the SVM. Features from logistic regression were only interpretable in linearly while random forest and XGBoost can be interpreted in both linear and non-linear ways. Moreover, XGBoost was modeled with raw data while random forest used imputed data; hence, features from XGBoost should be more persuasive.

Overall, logistic regression adopted features through stepwise selection procedure; random forest and XGBoost handled features automatically during model construction; SVM used selected features from XGBoost.

### Performance Evaluation

The testing performance of different models was evaluated by recall (sensitivity), specificity, Youden Index (Youden, 1950), accuracy, F1-score, and area under the curve (AUC) of receiver operating characteristics (ROC) in all three levels models. In attempt to maximize both the sensitivity and specificity of the fitted predictive model, the cut-off was chosen based on the Youden index, γ, which is derived from sensitivity and specificity and denotes a linear correspondence balanced accuracy, given as:

γ=sensitivity+specificity-1

The Youden index has been commonly used to evaluate predictive model performance and has shown good performance on model assessment. The best cut-off was obtained through the indication of largest Youden index based on a 10-fold cross-validation.

## Results

Among the total 300 patients, there were 160 females and 140 males aged from 27 to 104 (mean 76.0 with standard deviation 13.4). LVO was present in 130 (43.3%) patients. Statistical summaries and naïve test were applied to investigate the relationships between single feature and LVO (Tables 1A,B). A wide range of factors were found having associations with an increased risk of LVO. *P*-values were obtained by using Student’s *t*-test for continuous variables, Fisher’s exact test, and Pearson’s chi-square test for categorical variables. Based on 1% significance level, dependent features from Level-1 included patients’ age, gender, limb weakness, facial weakness, and left- and right-facial weakness; while Level-2 dependent features included patients’ habit, e.g., smoking, pre-existing medical condition, e.g., atrial fibrillation, clinical test scales at the time of onset, e.g., GCS and its corresponding subscales including eye, verbal, and motor function.

Besides, among the 300 patients, 74 patients had hyperdense MCA signs on their CT brain scans and 68 (97.1%) of them had LVO; while among the remaining 224 patients without hyperdense MCA sign, only 62 (27.4%) of them suffered from LVO (*p*-value: < 2.20e-16). Hence, the presence of the MCA signs is associated with LVO, but not all LVO patients had hyperdense MCA signs on their CT scans.

The models’ performance in the testing cohort is shown in Table 2. The cut-offs were obtained through the indication of largest Youden index based on a 10-fold cross-validation under training cohort. In Level-1, the best result was obtained by XGBoost method with Youden index of 28.3%, accuracy of 64.0%, recall (sensitivity) of 65.1%, specificity of 63.2%, F-score of 0.609, and AUC of 0.686, respectively. In Level-2, the best result was obtained by SVM with Youden index of 59.1%, accuracy of 78.0%, recall (sensitivity) of 90.7%, specificity of 68.4%, F-score of 0.780, and AUC of 0.839, respectively. In the Level-3 model, the XGBoost method achieved best results, where the Youden index of 63.8%, accuracy of 80.0%, recall (sensitivity) of 95.3%, specificity of 68.4%, F-score of 0.804, and AUC of 0.847, respectively.

**TABLE 2:** Models Performance under Level-1, -2, and -3.

	Data	Models	Youden	Accuracy	Recall	Specificity	*F*-score	AUC
Level-1	Raw	Logistic regression	22.4%	61.0%	62.8%	59.6%	0.581%	0.699%
		Random forest	23.6%	61.0%	67.4%	56.1%	0.598%	0.675%
		SVM	24.3%	64.0%	48.8%	75.4%	0.538%	0.682%
		XGBoost	**28.3%**	64.0%	65.1%	63.2%	0.609%	0.686%
Level-2	Imputed	Logistic regression	41.7%	71.0%	69.8%	71.9%	0.674%	0.789%
		Random forest	50.3%	73.0%	90.7%	59.6%	0.742%	0.816%
		SVM	**59.1%**	78.0%	90.7%	68.4%	0.780%	0.839%
	Raw	XGBoost	57.9%	77.0%	93.0%	64.9%	0.777%	0.809%
Level-3	Imputed	Logistic regression	53.2%	75.0%	88.4%	64.9%	0.752%	0.843%
		Random forest	55.6%	76.0%	90.7%	64.9%	0.765%	0.870%
		SVM	53.9%	76.0%	83.7%	70.2%	0.750%	0.827%
	Raw	XGBoost	**63.8%**	80.0%	95.3%	68.4%	0.804%	0.847%

*Boldfaced Youden index means the best results achieved within specific level of modeling.*

Comparing all four machine learning algorithms, the XGBoost method gave robust and accurate performance in all three levels. Though SVM had slightly better result in Level-2, its performance was unsatisfying in Level-3; besides, the SVM required selected variables derived from XGBoost. More importantly, instead of using imputed data, the XGBoost method took the raw data as input, which avoid the risk of inaccurate imputation.

**FIGURE 5:**
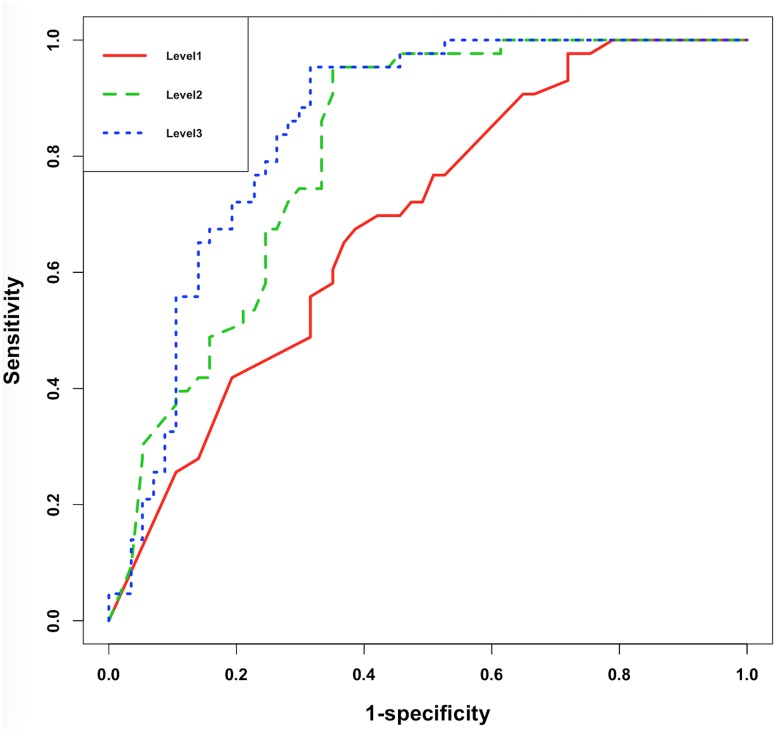
ROC curve of XGBoost models under Level-1, -2, and -3.

The receiver operator characteristic (ROC) curve (Figure 5) showed significant improvement of performance in XGBoost models between each pair of Levels, indicating that the additional features did assist model building.

**FIGURE 6:**
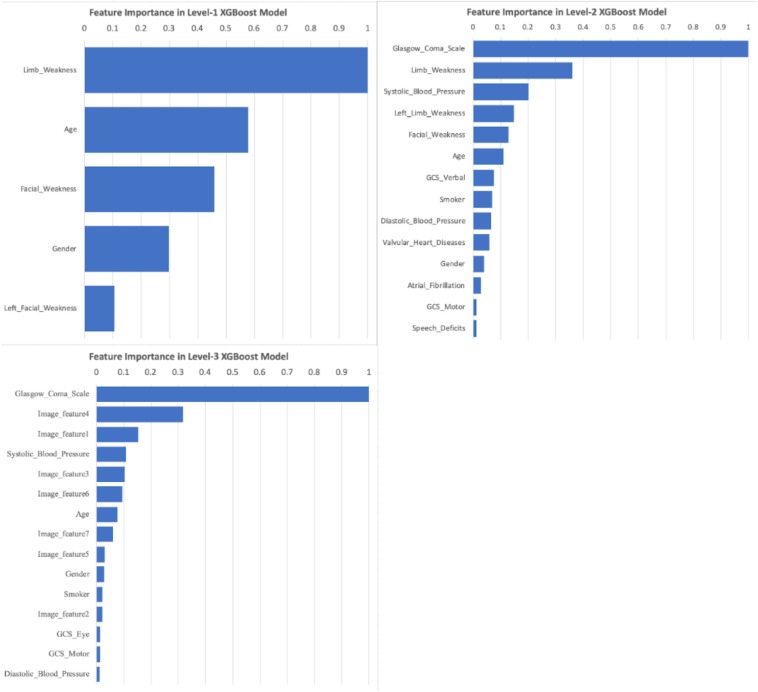
Important Features of XGBoost models under Level-1, -2, and -3. Feature importance scores were calculated based on the total gain of all features within XGBoost models. The scales were standardized based on the largest gain (scaled to 1.0), and then sorted. Any scaled importance scores less than 0.01 were omitted.

As XGBoost method can automatically identify the relative importance of variables, we first converted the total gain of the most important feature to 1 and then standardized all other features based on it. By sorting the scales, Level-1 took the limb weakness as the most important feature; while the GCS ranked as the top feature in both Level-2 and Level-3 (Figure 6). Image features from CT scans took considerable proportion of important features in Level-3, implying their contribution was substantial.

## Discussion

Acute ischemia stroke patients with LVO had high morbidity and high mortality rate, and close to one-third of patients passed away within 30 days of admission based on a research with population in Hong Kong (Tsang et al., 2019b). As the benefit of EVT for LVO diminishes over time, streamlining prehospital diagnosis and triage plays a vital role in improving clinical outcome by reducing prehospital and interhospital delays. Hence, a rapid identification of potential LVO patients in prehospital stage can effectively triage patients to appropriate stroke hospitals, thereby avoiding interfacility transfers or overburdening the primary stroke department with non-EVT-eligible patients.

The innovation of our study, on one hand, is the construction of an automated evaluation system based on a hierarchy of data easily accessible by prehospital assessment on ambulance and the early triage phase of clinical evaluation in emergency departments. On the other hand, unlike most previous research studies, our method is capable of combining structured demographic and clinical data with non-structured CT imaging data. The present model can potentially aid prehospital stroke triage (Level-1 and -2) and assist in the early activation of EVT treatment pathways in the stroke hospital immediately when NCCT is performed (Level-3).

The deep learning architecture in our study worked as a feature extractor trained with the task of MCA segmentations. The hyperdense MCA sign is a biomarker for LVO, and their presence largely implies the potential risk of LVO. Since all our NCCT were 5 mm thick-cut scans and the number of valuable slices was only six per patient; we had to adopt a 2D architecture rather than 3D. Second, the feature extractor needed to provide unbiased features for both training and testing cohort. If we use the deep learning architecture to directly predict LVO, instead of hyperdense MCA sign, the training cohort would be overfitting and the subsequent machine learning algorithms would face similar problems when using these features as well. Patients without hyperdense MCA sign can still be suffering from LVO stroke. In fact, most false negative patients within Level-2 did not demonstrate MCA sign on their CT scans; thereby, no valuable information can be extracted to correct those misclassified subjects. As a result, the improvement of model performance was less apparent on Level-3 when compared with Level-2.

Among all four implemented machine learning methods, XGBoost was the top choice for future applications. Apart from its outstanding performance, one key advantage was its ability to handle missing values in both training and prediction. Missing value is an inevitable problem when dealing with data obtained from retrospective clinical database. Unfortunately, most research studies were launched with the presumption of the data being complete. Focusing only on patients with complete data attributes would cause biased results, and eliminating incomplete attributes or subjects containing missing values might cause the loss of critical information as well. Though logistic regression, random forest, and SVM methods can be done with imputed dataset, they cannot be deployed if the upcoming new observation contains any missing data. In such cases, training data are required for imputation references, which is undesirable.

A recent systematic review of pre-hospital LVO diagnostic instruments such as NIHSS, CPSSS, LAMS, and RACE found the AUCs of these scales were in the range of 0.70–0.85. While these scales may achieve either a high recall or high specificity, none was capable to provide both (Smith et al., 2018). Most of these instruments were based on clinical signs detected by physical examination but did not consider patient-specific medical background and stroke risk factors. Another study by Chen et al. (2018) compared the results obtained by NIHSS with the addition of clinical features modeled with artificial neural networks, and their results indicated the additional clinical features would enhance the model’s performance. While their results were comparable with the present model, the use of NIHSS in their model limited its utility in the pre-hospital setting as it requires detailed neurological examination including visual field, ataxia, sensation, and attention assessment. Our model using the simple Face Arm Speech test with clinical features is less time-consuming to perform and does not require advanced training for ambulance or triage staff, thereby may be better suited for rapid LVO diagnosis and triage.

There are several limitations to this study. First, the NCCT brain scans were 5 mm thick-cut, and subtle hyperdense MCA sign may not be identified. Besides, the thick-cut CT scans limited our deep learning architecture to 2D design. Second, our model is based on retrospective data, and further prospective validation in populations of other ethnicity is needed to assess its generalizability. Third, the diagnosis of LVO stroke was made based on the clinical evolution and follow-up imaging of the patients, and only a small proportion of the study cohort (<5%) had angiogram within the acute setting. This nevertheless reflected the clinical utility of the algorithm in resource-tight healthcare systems where advanced neuroimaging may not be readily available (Tsang et al., 2019a). Finally, a larger patient cohort may improve the performance of the deep learning model for NCCT imaging.

## Conclusion

In this study, we established a three-tier diagnostic tool using machine learning for acute LVO stroke, based on a hierarchy of demographic, clinical, and imaging data. The Level-1 model provided preliminary triage for emergency dispatchers and required only basic demographic information and easily observable symptoms. In the Level-2 model, additional medical history and patients’ vital signs were utilized to provide rapid and accurate LVO diagnosis, potentially allowing for direct ambulance transfer to EVT hospitals capable of providing optimal care for LVO stroke patients. The inclusion of NCCT brain scans, obtainable from emergency departments, in the Level-3 model further enhanced the specificity of LVO diagnosis and may streamline the treatment pathway for acute reperfusion therapies.

To the best of our knowledge, this is the first study that combined structured clinical data with non-structured CT imaging data. Comparing with previous studies, our model achieved superior performance and can potentially improve pre-hospital triage systems for AIS.

## Data Availability Statement

The data were under the control of the Hong Kong Hospital Authority and were released to the authors for study on the condition that these data would not be shared with persons outside the study team. Reasonable request for data access can be made to the Hong Kong Hospital Authority and such requests will be adjudicated on a case-by-case basis.

## Ethics Statement

The studies involving human participants were reviewed and approved by the local Institutional Review Board (The University of Hong Kong/Hospital Authority Hong Kong West). The ethics committee waived the requirement of written informed consent for participation.

## Author Contributions

AT, ET, GL, and PY conceived and designed the study. ET and GL coordinated the study. JY and PY contributed to the literature search. PW and CL collected and anonymized the data. AT and JY read the discharge notes and labeled the ground truth. JY contributed to data analysis and interpretation under the supervision of PY. JY wrote the first draft of the article, which was then critically revised and approved by all authors.

## Conflict of Interest

The authors declare that the research was conducted in the absence of any commercial or financial relationships that could be construed as a potential conflict of interest.
